# Hydromorphone ameliorates postoperative pain and depressive disorder in women receiving cesarean section under spinal anesthesia

**DOI:** 10.3389/fmed.2025.1604600

**Published:** 2025-10-20

**Authors:** Rongyi Ouyang, Jinming Jiang, Wenqiang Li, Fu He, Libin Yang, Xianbing Kou, Junchao Dai, Mingliang Xu, Yulin Liu, Jian Zhan

**Affiliations:** ^1^Department of Anesthesiology, The Affiliated Hospital, Southwest Medical University, Luzhou, China; ^2^Department of Anesthesiology, Wangcang People's Hospital, Guangyuan, China; ^3^Department of Anesthesiology, The Fourth Affiliated Hospital of Southwest Medical University, Meishan, China; ^4^Anesthesiology and Critical Care Medicine Key Laboratory of Luzhou, The Affiliated Hospital, Southwest Medical University, Luzhou, China

**Keywords:** hydromorphone, sufentanil, postoperative depression, patient-controlled intravenous analgesia (PCIA), cesarean section

## Abstract

**Background:**

Pain and depressive mood disorders during cesarean sections negatively impact both mothers and infants. Studies have shown that hydromorphone has positive effects on both pain management and depressive mood disorders. This study examines how hydromorphone influences postoperative pain and depressive disorders in parturients undergoing cesarean sections under spinal anesthesia.

**Methods:**

This single-center, controlled, randomized trial involved 130 patients. Parturients in the intervention (H) group received patient-controlled intravenous analgesia (PCIA) with hydromorphone combined with sufentanil, while those in the control (S) group received sufentanil alone. All cesarean sections were performed under spinal anesthesia. Postoperative pain scores at rest and during movement were assessed using the Visual Analog Scale (VAS) at 4 h (T0), 8 h (T1), 12 h (T2), 24 h (T3), and 48 h (T4) postoperatively. The Beck Depression Inventory (BDI) and Beck Anxiety Inventory (BAI) were utilized to measure depressive and anxiety disorders at 3 days and 6 weeks postoperatively. Adverse reactions were also recorded.

**Results:**

The H group had significantly lower resting VAS scores at all postoperative time points (*p* = 0.008) and significantly lower movement VAS scores (*p* < 0.001) compared to the S group. At 3 days postoperatively, BDI scores showed no statistically meaningful variations (*p* = 0.057) or BAI scores (*p* = 0.444) between the two group. However, at 6 weeks postoperatively, the H group had significantly lower BDI scores (*p* = 0.001) and BAI scores (*p* = 0.012). No statistically significant differences in operative time were observed between the groups. (*p* = 0.086), time to first ambulation (*p* = 0.092), sleep quality scores (*p* = 0.132), or adverse reactions, including chills (*p* > 0.999), pruritus (*p* = 0.109), nausea and vomiting (*p* = 0.718), respiratory depression (*p* = 0.619), or dizziness (*p* = 0.619).

**Conclusion:**

The synergistic use of hydromorphone and sufentanil in PCIA provides superior analgesia for postoperative pain and decreases postoperative depression and anxiety scores in parturients undergoing cesarean sections.

## 1 Introduction

In China, the cesarean section rate reached 36.7% in 2018 (1). The cesarean section is a widely utilized mode of delivery in obstetric practice (2). Surgical incisions and uterine contractions can cause severe postoperative pain in parturients, significantly affecting postpartum recovery and mother-infant interaction (3). Studies have shown that acute perinatal pain is a key factor contributing to postpartum depressive disorders (4–6), with the incidence of depressive disorders in Chinese parturients after cesarean section reaching as high as 22% (7). Furthermore, depressive disorders can impact the long-term prognosis of parturients, not only affecting their health and quality of life, but also potentially influencing child development and parenting behaviors (8–10). Therefore, effective strategies are needed to manage postoperative pain and depressive disorders in parturients.

Patient-controlled intravenous analgesia (PCIA) is a widely employed technique for postoperative pain management in patients undergoing cesarean section, providing effective pain control and high patient satisfaction (11, 12). Opioid analgesics consistently demonstrate unparalleled efficacy in treating moderate-to-severe pain and are commonly used in PCIA (11, 13). Hydromorphone (HM) is a semi-synthetic opioid analgesic that primarily acts on *μ*-receptors and partially on *δ*-receptors. It offers advantages such as potent analgesia, rapid onset, and fewer adverse effects (14). HM exerts its analgesic effects by activating μ-receptors and has an analgesic potency 5 to 10 times that of morphine, similar to sufentanil, allowing for effective titration and pain control (15, 16). Its inactive metabolites makes it a safe and effective analgesic (17). Two randomized studies have shown that HM effectively controls postoperative pain in cesarean sections and other surgeries (18, 19).

In addition to activating *μ*-receptors, HM also partially activates *δ*-receptors, which are involved in regulating chronic pain, anxiety, and depressive responses (20–22). Rapp et al. reported that compared to morphine, HM improved mood in patients undergoing lower abdominal surgery, while both drugs provided adequate analgesia with no difference in side effects (23). However, studies on the application of HM in postoperative depressive disorders in patients undergoing cesarean section remain limited and inconclusive (24). Only two randomized controlled trials have suggested a correlation between HM-based PCIA and postoperative depressive disorders in patients undergoing cesarean section (24, 25).

Therefore, we sought to systematically investigate the impact of HM on postoperative pain relief and depressive disorders in patients receiving cesarean section by comparing HM combined with sufentanil PCIA versus sufentanil-only PCIA. The study will assess resting and movement visual analog scale (VAS) scores, Beck Depression Inventory (BDI) scores, Beck Anxiety Inventory (BAI) scores, operative time, time to first ambulation, sleep quality scores, and adverse reactions.

## 2 Methods

This was a single-center, randomized, controlled trial conducted at Wangcang People’s Hospital in Sichuan Province, China. The research was conducted in compliance with the CONSORT guidelines for reporting clinical trials.

### 2.1 Ethics, consent, and permissions

The Ethics Committee of Wangcang People’s Hospital approved the study protocol (No. 23–006). The study adhered to the Declaration of Helsinki and was registered in the Chinese Clinical Trial Registry (ChiCTR2300068741). Informed consent was obtained from all eligible participants before enrollment.

### 2.2 Participants

Between February 2023 and September 2023, pregnant women undergoing their first cesarean section under spinal anesthesia and proficient in the Chinese language were recruited. The eligibility criteria consisted of participants aged 20 to 35 years who had an American Society of Anesthesiologists (ASA) physical status I or II. The exclusion criteria comprised individuals with a documented psychiatric disorder history, active psychotropic medication use; any record of substance abuse involving alcohol, illicit drugs, opioid analgesics; current treatment with monoamine oxidase inhibitors; severe cardiovascular, cerebrovascular, hepatic, renal, or hematopoietic system diseases; and allergic predisposition or known allergies to opioids or HM. Participants were randomly assigned in a 1:1 ratio to either the intervention or control group using computer-generated randomization. The allocation process utilized a computerized randomization system to equally distribute participants (1:1 ratio) between the intervention (H) group and the control (S) group.

### 2.3 Anesthesia procedure and analgesia protocol

Parturients were instructed to fast for 8 h and refrain from drinking for 4 h prior to surgery. Upon entering the operating room, they were monitored based on electrocardiography (ECG), oxygen saturation (SPO_2_), respiratory rate (RR), and noninvasive blood pressure (NIBP), and received continuous oxygen via a facemask (2.0–3.0 L/min). An intravenous (IV) line was set up in the upper limb. The parturients were then placed in the left lateral position and routine disinfection and draping were performed. Subarachnoid puncture was performed at the L3–4 interspace, and after confirming cerebrospinal fluid outflow, 15 mg of 0.5% ropivacaine (Zhejiang Xianju Pharmaceutical Co., Ltd., specification: 10 mL: 100 mg) was administered, ensuring that the sensory block level remained below T6. No serious adverse events occurred during surgery in either group. The postoperative analgesia protocol was double-blinded, with anesthesiologists, participants, and follow-up personnel blinded to group assignments. Intervention Group (H): Immediately after fetal delivery, hydromorphone (10 μg/kg, concentration: 0.5 mg/mL) was administered intravenously within 1 min. The postoperative PCIA regimen consisted of hydromorphone (Yichang Humanwell Pharmaceutical Co., Ltd., specification: 2 mL: 2 mg) 0.2 mg/kg + sufentanil 1.5 μg/kg + tropisetron (5 mg). Control (S) Group: Immediately after fetal delivery, sufentanil (Yichang Humanwell Pharmaceutical Co., Ltd., specification: 1 mL: 50 μg) 8 μg, concentration: 5 μg/mL, was intravenously injected within 1 min. The postoperative PCIA formula consisted of sufentanil 3 μg/kg + tropisetron 5 mg.

The PCIA solution was reconstituted with 0.9% sodium chloride to standardize the pharmaceutical preparation at 100 mL total volume, programmed with a 2 mL/h background infusion, 1 mL on-demand bolus availability, and a 15-min safety interval between consecutive bolus administrations, lasting for 48 h. Before leaving the operating room, the anesthesiologist assessed the parturients’ consciousness and vital signs to ensure safety, after which they were transferred to the ward.

### 2.4 Observation indicators

#### 2.4.1 Primary indicators

Postoperative pain was evaluated using static and dynamic Visual Analog Scale (VAS) scores at five time points: 4 h (T0), 8 h (T1), 12 h (T2), 24 h (T3), and 48 h (T4). The VAS scores range from 0 to 10, where 0 signifies no pain, 1–3 indicates mild pain, 4–6 represents moderate pain, and 7–10 denotes severe pain. The Beck Depression Inventory (BDI) was utilized to evaluate the participants’ depression states. The Beck Anxiety Inventory (BAI) was employed to evaluate participants’ anxiety states.

#### 2.4.2 Secondary indicators

Surgical time and time to first ambulation were recorded. Sleep quality scores were assessed on the night of surgery and on the first and second days after surgery. The sleep quality scoring criteria were good sleep (1 point), restless sleep (2 points), nightmares (3 points), and insomnia (4 points).

Adverse reactions related to PCIA, including chills, pruritus, nausea, vomiting, respiratory depression, and dizziness, were observed and recorded.

### 2.5 Sample size estimation

Postoperative pain scores were used as primary outcome variables. Based on previous studies, the average VAS score for patients undergoing cesarean section was 4.3 ± 0.97 (26). It was estimated that the H group would reduce the VAS score by a minimal clinically significant difference (MCID) of 0.5 point compared to the S group. Using a significance level (*α*) of 0.05, power of 0.90, repeated measurements (M) = 5, standard deviation (*σ*) set at 0.97 based on reference data, and a conservative autocorrelation coefficient (*ρ*) of 0.7, the sample size was determined using PASS 15.0 for a repeated-measures design. Each group was estimated to have 61 participants. Considering a 5% dropout rate, the minimum required sample size was 65 by group, resulting in a total of 130 participants.

### 2.6 Statistical analysis

The data were analyzed using SPSS version 22 from IBM Inc., Armonk, NY, USA. Categorical variables were presented as numbers with percentages. To minimize the influence of potential confounding factors on the results between groups, independent t-tests were conducted to compare demographic data, surgical time, and time to first ambulation. Repeated-measures ANOVA and Wilcoxon rank-sum tests were employed to analyze the static and dynamic VAS scores, sleep quality, BDI, and BAI scores at different time points within and between groups. The chi-squared test was employed to compare the incidence of postoperative adverse reactions among the groups.

## 3 Results

**Figure 1:**
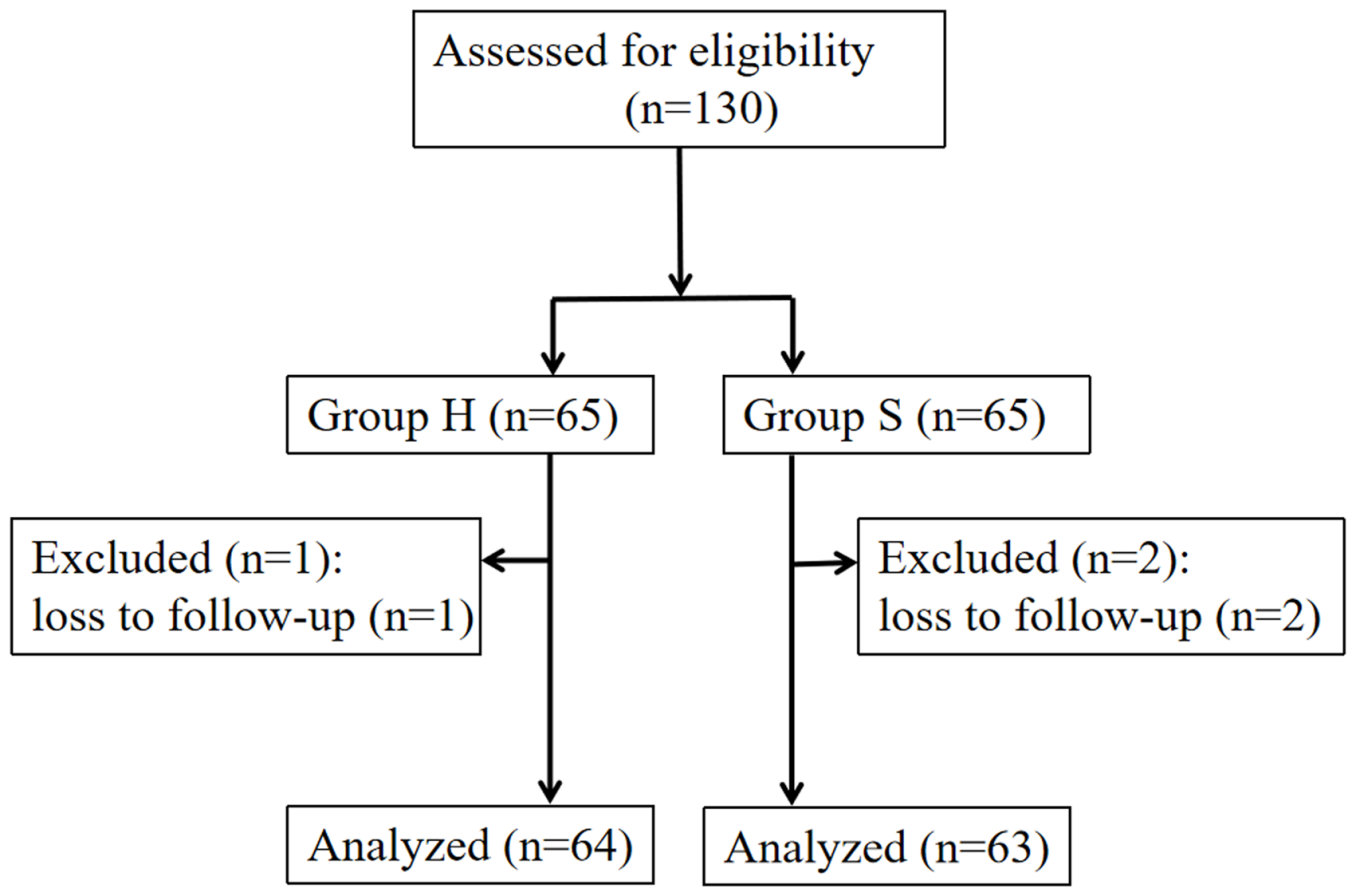
Schematic illustration of the enrolled patient.

A total of 3 out of 130 participants were lost to follow-up at 6 weeks postpartum, resulting in 127 participants included in the final analysis (Figure 1). The H group had an average age of 28.6 ± 3.7 years and a body mass index (BMI) of 27.3 ± 3.6 kg/m^2^, while the S group had an average age of 28.3 ± 3.8 years and a BMI of 27.9 ± 2.6 kg/m^2^. There were no statistically significant differences in age or BMI between the groups (*p* > 0.05).

### 3.1 Comparative analysis of pain relief between the two groups

The differences in postoperative resting and movement VAS scores between groups were statistically significant (Table 1).

**Table 1:** Postoperative resting and movement VAS scores between groups.

Group	*n*	Resting VAS scores	Movement VAS scores
H	64		
T_0_		1.031 ± 0.816	2.78 ± 0.49
T_1_		1.047 ± 0.844	2.80 ± 0.54
T_2_		1.031 ± 0.816	2.80 ± 0.51
T_3_		1.141 ± 0.774	2.86 ± 0.50
T_4_		1.141 ± 0.774	2.81 ± 0.50
S	63		
T_0_		1.413 ± 0.638	3.17 ± 0.46
T_1_		1.397 ± 0.636	3.17 ± 0.46
T_2_		1.413 ± 0.638	3.14 ± 0.43
T_3_		1.397 ± 0.583	3.16 ± 0.41
T_4_		1.429 ± 0.588	3.19 ± 0.43

The *p*-value for the movement VAS score was 0.008, while the *p*-value for the resting VAS score was <0.001.

### 3.2 Comparison of Beck Depression Inventory (BDI) scores between groups

There was a statistically significant difference in depression scores between the H group and the S group. An interaction effect was noted between group and time. Pairwise comparisons revealed no significant differences in depression scores between groups preoperatively or 3 days postoperatively. However, a statistically significant difference was observed 6 weeks postoperatively (Tables 2, 3).

**Table 2:** Generalized Estimating Equation (GEE) analysis results of Beck Depression Inventory (BDI) scores between the two groups.

Variables	Wald chi-square	df	*P*
Group	7.368	1	0.007
Time	63.561	2	<0.001
Group * Time	6.558	2	0.038
Intercept	90.155	1	<0.001

**Table 3:** Comparison of subgroup analysis of Beck Depression Inventory (BDI) scores at various time points between the two groups.

Time	(I) group	(J) group	Mean difference (I-J)	Std. Error	*p-*value	95% CI for Wald difference	
						Lower bound	Upper bound
Preoperative	S	H	0.75	0.395	0.058	−0.03	1.52
	H	S	−0.75	0.395	0.058	−1.52	0.03
3 days after surgery	S	H	1.12	0.654	0.086	−0.16	2.40
	H	S	−1.12	0.654	0.086	−2.40	0.16
6 weeks after surgery	S	H	3.13	1.096	0.004	0.99	5.28
	H	S	−3.13	1.096	0.004	−5.28	−0.99

### 3.3 Comparison of Beck Anxiety Inventory (BAI) scores between the two groups

Intergroup comparison revealed no statistically significant differences in BAI scores both at baseline (*p* = 0.202) and on postoperative day 3 (*p* = 0.444). However, at 6 weeks postoperatively, the BAI scores in the H group were substantially lower than those in the S group, with a significant difference (*p* = 0.012), as shown in Table 4.

**Table 4:** Beck Anxiety Inventory (BAI) scores in the two groups.

	H group (*n* = 64)	S group (*n* = 63)	Statistical parameters	*P-*value
Preoperative	2.00 (0.00,4.00)	2.00 (1.00,5.00)	*Z* = –1.276	0.202
3 days after surgery	2.00 (0.00,5.75)	2.00 (0.00,5.00)	*Z* = –0.766	0.444
6 weeks after surgery	0.50 (0.00,4.00)	3.00 (0.00,7.00)	*Z* = –2.527	0.012

### 3.4 Comparison of surgical time and time to first ambulation between the two groups

No statistically significant differences were detected in the surgical time (*p* = 0.086) or time to first ambulation (*p* = 0.092) between groups (Table 5).

**Table 5:** Surgical time and time to first ambulation in the two groups.

Group	*n*	Surgical time(min)	Time to first ambulation (min)
H	64	50.09 ± 8.46	25.70 ± 3.98
S	63	50.95 ± 7.27	26.92 ± 4.10

### 3.5 Comparison of sleep quality scores between groups

No significant difference was observed in the sleep quality scores between groups (*p* = 0.132), as shown in Table 6.

**Table 6:** Sleep quality scores in the two groups.

Group	*n*	Sleep quality scores
H	64	
The night after surgery		1.266 ± 0.542
One day after surgery		1.188 ± 0.393
Two days post-surgery		1.156 ± 0.366
S	63	
The night after surgery		1.159 ± 0.368
One day after surgery		1.095 ± 0.296
Two days post-surgery		1.079 ± 0.272

### 3.6 Comparison of postoperative complications between the two groups

No significant differences were observed in the incidence of postoperative complications between groups (Table 7).

**Table 7:** Comparison of postoperative complications in the two groups.

Group	*n*	Chills	Pruritus	Nausea and vomiting	Respiratory depression	Dizziness	Total adverse events
H	64	4 (6.3%)	3 (4.7%)	3 (4.7%)	1 (1.6%)	3 (4.7%)	8 (12.5%)
S	63	3 (4.8%)	8 (12.7%)	4 (6.3%)	2 (3.2%)	1 (1.6%)	15 (23.8%)
*P-*value		>0.999	0.109	0.718	0.619	0.619	0.098

## 4 Discussion

In summary, our study found that, compared to sufentanil PCIA, HM combined with sufentanil PCIA was associated with lower depression and anxiety scores at 6 weeks postoperatively. However, no significant differences were observed in depression and anxiety scores at 3 days postoperatively, and the differences in resting and movement VAS scores, though statistically significant, were not clinically meaningful.

Opioids are among the most commonly used drugs for postoperative analgesia after cesarean section (27). Both HM and sufentanil act on *μ*-receptors and are widely used for postoperative pain management. HM’s metabolites are inactive, making it a safe and effective analgesic (17, 28–30). Prior studies have demonstrated the efficacy of HM in PCIA following cesarean section, offering similar analgesic efficacy to sufentanil with fewer adverse effects (15). Yang et al. found no significant differences were observed in resting and movement VAS scores between patients undergoing radical colorectal cancer surgery who received HM PCIA and those who received sufentanil PCIA (13). Similarly, there were no significant differences in postoperative VAS scores between patients receiving HM combined with sufentanil PCIA and those receiving sufentanil alone (31). In line with these findings, our study showed that HM combined with sufentanil PCIA resulted in significantly lower resting and movement VAS scores at all time points compared to sufentanil PCIA. However, the differences were less than 1 point, rendering them clinically insignificant. In contrast, HM was more effective than sufentanil for PCIA in children undergoing congenital structural deformity repair (32). However, that study used the FLACC pain scale, whereas our study employed the VAS scale, making direct comparisons difficult. In our study, both groups had resting and movement VAS scores indicative of mild pain, suggesting that early postoperative acute pain was effectively managed.

Post-cesarean section pain is closely linked to postpartum depression. Increased postoperative pain following a cesarean section raises the risk of postpartum depression, with a 6% higher risk within 6 months postpartum compared to beyond 6 months (33–36). The global incidence of postpartum depression is estimated to be 10–20%, and it can persist for up to 2 years, with approximately 40% of affected women experiencing recurrence after subsequent pregnancies (37, 38). Therefore, an ideal postpartum opioid analgesic should not only provide effective pain relief but also help alleviate postoperative anxiety and depression while minimizing physiological side effects. In addition to acting on *μ*-receptors, HM partially acts on *δ*-receptors. Previous studies have confirmed that δ-receptor agonists can be used to treat anxiety and depressive disorders (21, 22, 39). Compared to morphine, HM has demonstrated an improvement in emotional responses among patients undergoing lower abdominal surgery (23). Yang et al. did not report any significant differences in anxiety and depression scores at 48 and 96 h postoperatively between patients with colorectal cancer receiving HM PCIA and those receiving sufentanil PCIA (13). Our study did not identify any significant differences in baseline anxiety and depression scores between the H and S groups. Although HM PCIA did not improve anxiety or depression scores at 3 days postoperatively, it significantly reduced these scores at 6 weeks postoperatively. Given that the incidence of depression within 5 days after cesarean section is 22.7% and can persist for up to 1 year postpartum (40, 41), the significant reduction in postpartum depression at 6 weeks in the H group may be related to the antidepressant effect of HM through the activation of *δ*-receptor.

In our study, there were no significant differences in age, BMI, surgical time, or time to first ambulation between the two groups. Liu et al. did not identify any significant difference in the time to first ambulation between patients who received HM combined with sufentanil PCIA and those who received sufentanil alone (31). Additionally, we compared postoperative sleep quality between the two groups. Previous studies have shown that opioids can enhance sleep quality while providing pain relief (42, 43). HM significantly alleviated pain and enhanced sleep quality among patients (44). Similarly, HM PCIA significantly enhanced pain management and sleep quality in patients suffering from postherpetic neuralgia (45). Consistent with these findings, our study found no sleep disturbances in either group, likely due to the effective pain relief and sleep-enhancing effects of both HM and sufentanil. Thus, HM offers multiple benefits, including analgesia, anxiety relief, depression relief, and improved sleep quality, making it a valuable opioid for clinical use.

While effective for pain relief, opioids may induce adverse effects, including nausea, vomiting, pruritus, and respiratory depression (13, 28, 32). HM PCIA was associated with higher incidences of pruritus and nausea compared to sufentanil PCIA, but no significant differences were observed between the two groups in terms of vomiting, respiratory depression, or dizziness (13). Similarly, in our study, there were no statistically significant differences in the incidence of vomiting, respiratory depression, or dizziness between the H and S groups, and the overall incidence of postoperative complications was similar. However, HM PCA demonstrated lower incidences of nausea, vomiting, drowsiness, and pruritus compared to sufentanil PCA (28). In our study, the similar rates of nausea, vomiting, pruritus, respiratory depression, and dizziness between the H and S groups may be attributed to the reduced concentration of HM when combined with sufentanil. Therefore, further research is required to investigate various concentrations of HM combined with sufentanil for postoperative analgesia to minimize adverse effects.

This study was a pilot investigation into the use of HM for alleviating depression and pain in parturients after spinal anesthesia for cesarean section. However, it possessed certain limitations. First, the limited sample size could lead to potential bias, necessitating larger studies. Second, we did not explore different concentration gradients of HM combined with sufentanil PCIA. Further research should aim to determine the optimal concentration that maximizes analgesic effects while minimizing side effects.

In conclusion, research on alleviating postoperative depressive disorders in patients undergoing cesarean sections remains limited. This study demonstrates that hydromorphone combined with sufentanil for PCIA is a safe and effective approach for reducing postoperative anxiety, depression, and acute pain. Subsequent research should concentrate on optimizing the dosage and combination of HM to better manage postoperative anxiety and depression in patients undergoing cesarean sections, providing new insights for clinical practice.

## Data Availability

The original contributions presented in the study are included in the article/supplementary material, further inquiries can be directed to the corresponding author/s.
